# Ciliated foregut cyst of the pancreas mimicking a cystic neoplasm

**DOI:** 10.1093/jscr/rjag885

**Published:** 2026-10-12

**Authors:** Wisal Al Sukaiti, Natasha Ranjit, Al Warith Al Hashmi, Salim Al Riyami

**Affiliations:** Independent Researcher, Manchester, United Kingdom; Independent Researcher, Manchester, United Kingdom; Hepatobiliary Surgery Department, The Royal Hospital, Al Ghubrah Street, Al Ghubrah, PO Box 1331, Muscat 111, Oman; Hepatobiliary Surgery Department, The Royal Hospital, Al Ghubrah Street, Al Ghubrah, PO Box 1331, Muscat 111, Oman

**Keywords:** case report, radiology, hepatobiliary surgery, ciliated foregut cyst, laparoscopic subtotal pancreatectomy

## Abstract

Pancreatic cystic lesions are being identified more frequently with widespread use of cross-sectional imaging. While most pancreatic cysts are mucinous neoplasms or pseudocysts, congenital cysts of foregut origin are exceptionally rare in this location, with these being commonly found in the mediastinum. Their occurrence in the hepatobiliary system is rare, and pancreatic involvement is even rarer. Fewer than 20 cases have been reported in literature since it was first described in the 1990s. Ciliated foregut cysts arise from abnormal development of the primitive foregut and, histologically, are lined by ciliated epithelium. In the pancreas, they may be asymptomatic and difficult to radiologically distinguish from other lesions, posing a diagnostic challenge. Given their rarity, reporting cases of ciliated foregut cysts of the pancreas adds valuable clinical insight. We present the case of a 67-year-old man with an incidentally discovered cyst in the body of the pancreas.

## Introduction

A 67-year-old man presented to our hepatobiliary surgery department with non-specific abdominal pain. As part of the initial assessment, a computed tomography (CT) scan of the abdomen was performed, which incidentally demonstrated a lobulated cystic lesion within the body of the pancreas measuring 6.5 × 5.1 × 4.6 cm. The rest of the pancreas, common bile duct, hepatic vessels, spleen, kidneys, adrenal glands, and bowel were normal. There was unrelated evidence of fatty infiltration of the liver and gallbladder distension.

He had a past medical history of type 2 diabetes mellitus.

## Case report

Following identification of the pancreatic cystic lesion, baseline laboratory investigations were performed as part of the evaluation. Routine blood tests, including full blood count, renal function, liver function tests, and coagulation profile, were within normal limits. Serum tumour markers were not elevated (CA19-9: 5 U/ml; CEA: 1.2 μg/l).

Initial contrast-enhanced CT of the abdomen demonstrated a cystic lesion in the body of the pancreas with features suggestive of a serous cystic neoplasm. The lesion was multiloculated with internal septations and lacked malignant features such as a solid component, mural nodules, diffusion restriction, or lymphadenopathy. However, given the cystic nature of the lesion and the need for further characterization, a pancreatic magnetic resonance imaging (MRI) was performed.

MRI of the pancreas with magnetic resonance cholangiopancreatography was undertaken using a pancreas-specific protocol, including dynamic post-contrast sequences. This demonstrated a lobulated, multiloculated cystic lesion within the pancreatic body measuring approximately 6.2 × 5.1 × 4.6 cm, which remained stable in size compared with prior imaging. The lesion showed high signal intensity on T2-weighted images and low signal intensity on T1-weighted images, without diffusion restriction. A faintly enhancing internal component was present. In addition, there was mild dilatation of the main pancreatic duct, measuring up to 3 mm, with possible communication between the duct and cystic lesion (Fig. 1). On post-contrast arterial-phase imaging, the lesion demonstrated faint enhancement of its internal septa (Fig. 2). These findings raised concern for an intraductal papillary mucinous neoplasm.

**Figure 1:**
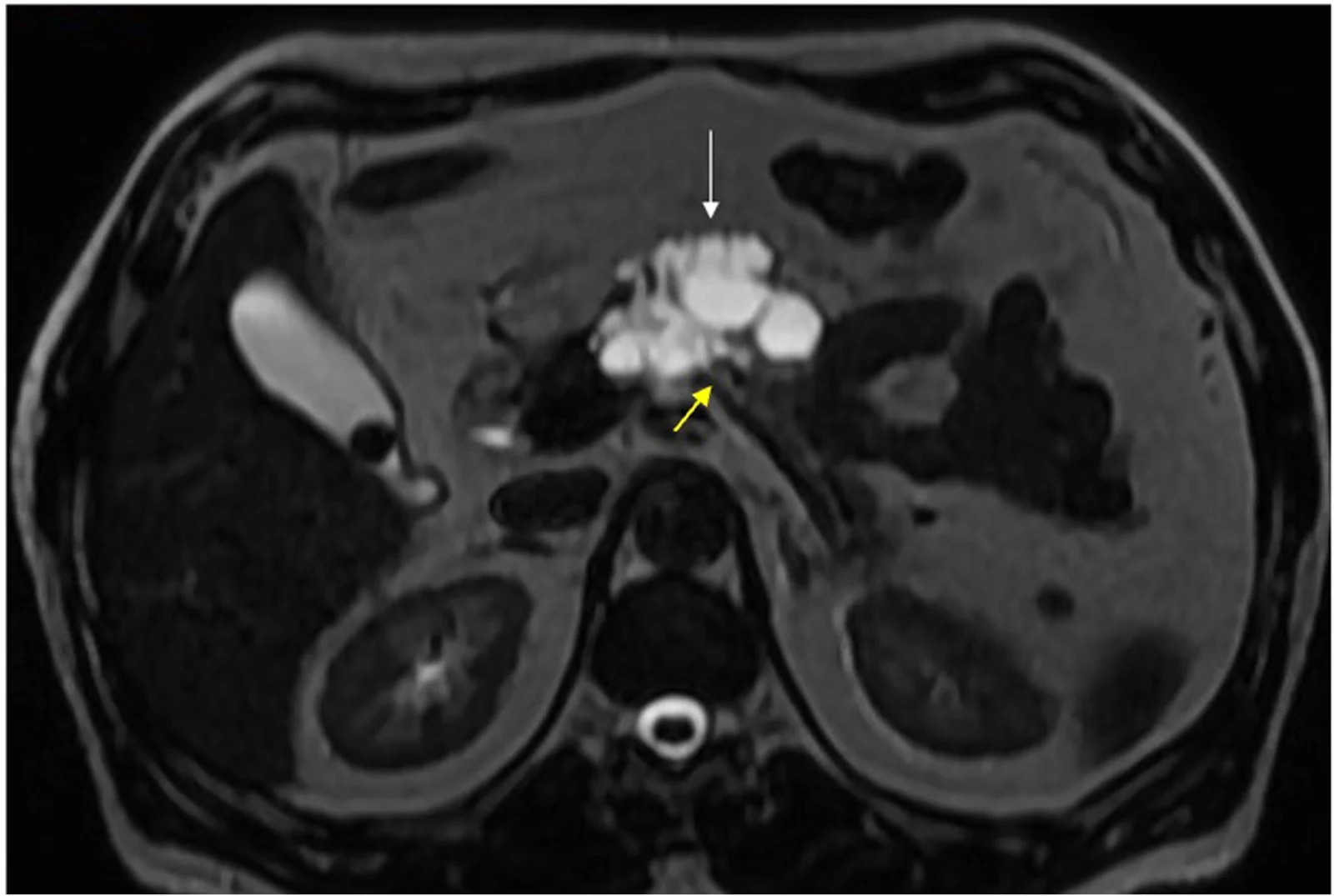
Axial T2-weighted MRI showing a lobulated, multiloculated cystic lesion in the pancreatic body (white arrow), with possible communication with the mildly dilated main pancreatic duct (yellow arrow).

**Figure 2:**
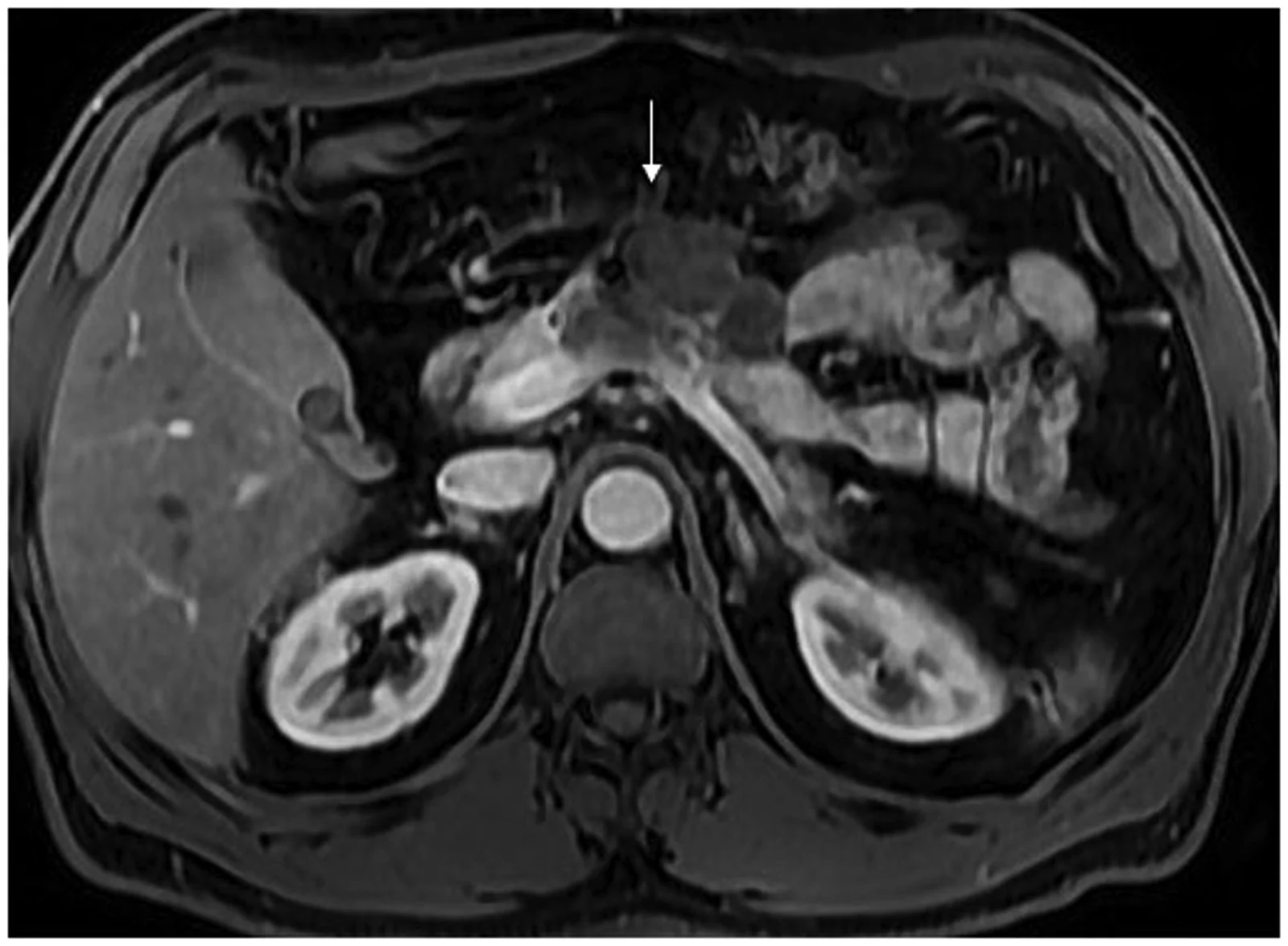
Axial T1-weight abdominal MRI showing a multiloculated cystic lesion in the pancreatic body with enhancing septa (arrow).

Endoscopic ultrasound was subsequently performed to further evaluate the lesion morphology and assess for high-risk features. This demonstrated two large multiloculated macrocystic lesions involving the pancreatic head and pancreatic neck/body regions. The cysts were septated, with no solid components or mural nodules identified. No convincing communication with the pancreatic duct was visualized. Fine-needle aspiration was performed using a 22-gauge needle, yielding ~15 ml of brownish cyst fluid for biochemical and tumour marker analysis. Cyst fluid CEA was within the normal range, while cyst fluid CA19-9 was elevated at approximately 300. These results were inconclusive for a mucinous cystic lesion when interpreted in isolation.

Despite a comprehensive multimodality assessment, pre-operative diagnostic uncertainty remained due to discordant findings across imaging modalities and cyst fluid analysis. Given the discrepant imaging findings regarding ductal communication and the absence of a definitive pre-operative diagnosis, a premalignant pancreatic cystic neoplasm could not be excluded. The patient, therefore, underwent laparoscopic subtotal pancreatectomy.

Intraoperative frozen section analysis of the proximal and distal pancreatic resection margins was undertaken to guide the extent of surgical resection. Both margins were negative for cyst epithelium and malignancy. Final histopathological examination confirmed a benign cystic lesion, subsequently diagnosed as a benign ciliated foregut cyst of the pancreas, with no evidence of dysplasia or invasive malignancy.

The postoperative course was complicated by a clinically insignificant pancreatic leak, from which the patient recovered well.

## Discussion

Foregut cystic abnormalities occurring outside the mediastinum, particularly within the hepatobiliary or pancreatic systems, are rare. They are thought to arise from sequestration of an outpouching of the foregut intended for respiratory differentiation [1]. Historically, a variety of terms have been used to describe cysts originating from the foregut, with distinctions based on location, embryological origin, epithelial lining, and wall characteristics. This case report specifically discusses ciliated foregut cysts, which are defined as cysts lined by pseudostratified ciliated columnar epithelium, potentially containing goblet cells and lacking additional distinguishing features, as seen in this case [1, 2].

A total of 18 cases in 17 case reports have been seen in the English literature since first reported by Kohzaki *et al.* in 1993. Case reports describing the presence of a foregut cyst within the pancreas have been compiled and presented in Table 1, with cyst characteristics and differential diagnoses recorded as originally described.

**Table 1:** Previous case reports of ciliated foregut cysts in the pancreas

Paper [Ref no.]	Age/gender	Inner epithelium	Size	Location	Presenting complaint
Kim *et al.* [1]	29/F	Pseudostratified ciliated columnar epithelium	41 × 35 mm and 16 × 11 mm	Tail	Incidentally finding
Roda *et al.* [15]	66/F	Epithelial lining with a ciliated border	58 × 38 × 48 mm	Tail	Incidental finding
Huang *et al.* [9]	45/F	Ciliated columnar epithelial cells	30 × 23 mm	Tail	Incidental finding
Dua *et al.* [13]	51/M	Pseudostratified tall ciliated columnar epithelium	69 × 24 mm	Body/tail	Abdominal pain, postprandial bloating, decreased appetite
Kawamoto [6]	66/M	Respiratory-like epithelium	27 mm	Body	Incidental finding
Munshi *et al.* [5]	33/M	Ciliated epithelium	54 × 43 × 21 mm	Uncinate process	Epigastric pain
Kohzaki *et al.* [3]	41/F	Ciliated simple columnar and pseudostratified epithelium	20 mm	Head	Epigastric pain
Vyas *et al.* [2]	52/F	Pseudostratified ciliated columnar epithelium	90 × 50 × 60 mm	Body/tail	Non-specific abdominal pain
Chen *et al.* [11]	47/F	Pseudostratified ciliated columnar epithelium	98 × 35 mm	Neck	Postprandial bloating
Bellevicine *et al.* [4]	50/F	Ciliated epithelium	19 × 9 mm	Head	Incidental finding
Woon *et al.* [14]	58/M	Pseudostratified ciliated epithelium	20 mm	Head	Abdominal pain
Huq *et al.* [10]	53/F	Ciliated epithelium	24 × 53 × 24 mm	Body	Incidental finding
Horky *et al.* [8]	46/M	Pseudostratified ciliated columnar epithelium	18 mm	Head	Jaundice, pruritis, dark urine, acholic stools
Wang *et al.* [7]	69/M	Ciliated respiratory-like epithelium	70 × 35 × 20 mm	Body/tail	Right upper quadrant discomfort
Zhang *et al.* [12]	54/F	Respiratory-like epithelium	30 × 30 × 10 mm	Body	Incidental finding

Most incidences were recorded in the body/tail of the pancreas and contained respiratory-like epithelium, specifically pseudostratified ciliated columnar epithelium. Cyst sizes varied across the literature, with the cyst described in this paper falling at the upper end of the reported spectrum. As was seen in this case, cysts initially identified on imaging were often thought to represent alternative lesions, such as intraductal papillary mucinous neoplasms, serous cystadenomas, or pseudocysts, and were not definitively diagnosed until excision and histopathological analysis.

This highlights the difficulty in diagnosing pancreatic ciliated foregut cysts based on imaging alone, as features including location, size, definition, locularity, and wall thickness demonstrate non-specific variation [3]. Fine needle aspiration findings have not been consistently described and reported aspirates range from milky white to brown viscous or clear serous fluid, limiting its diagnostic utility [2]. Histopathological analysis following excision remains the most reliable method for confirming the diagnosis [3].

Although ciliated foregut cysts are benign lesions, malignant transformation has previously been reported in other anatomical locations, such as the liver [12]. While malignant transformation has not been reported in pancreatic ciliated foregut cysts, the occurrence of malignancy in foregut cysts at other anatomical sites suggests that, in the presence of concerning clinical or radiological features, a prompt operative approach may be justified.
